# Diel spatio-temporal patterns of humpback whale singing on a high-density breeding ground

**DOI:** 10.1098/rsos.230279

**Published:** 2024-01-24

**Authors:** Anke Kügler, Marc O. Lammers, Adam A. Pack, Ludovic Tenorio-Hallé, Aaron M. Thode

**Affiliations:** ^1^ University of Hawai‘i at Mānoa, Honolulu, HI, USA; ^2^ Bioacoustics and Behavioral Ecology Lab, Syracuse University, Syracuse, NY, USA; ^3^ Oceanwide Science Institute, Honolulu, HI, USA; ^4^ Hawaiian Islands Humpback Whale National Marine Sanctuary, Kīhei, HI, USA; ^5^ University of Hawai‘i at Hilo, Hilo, HI, USA; ^6^ The Dolphin Institute, Hilo, HI, USA; ^7^ Marine Physical Laboratory, Scripps Institute of Oceanography, University of California, San Diego, La Jolla, CA, USA

**Keywords:** humpback whale, *Megaptera novaeangliae*, Hawaii breeding ground, diel habitat use, song, acoustic masking

## Abstract

Humpback whale song chorusing dominates the marine soundscape in Hawai‘i during winter months, yet little is known about spatio-temporal habitat use patterns of singers. We analysed passive acoustic monitoring data from five sites off Maui and found that ambient noise levels associated with song chorusing decreased during daytime hours nearshore but increased offshore. To resolve whether these changes reflect a diel offshore–onshore movement or a temporal difference in singing activity, data from 71 concurrently conducted land-based theodolite surveys were analysed. Non-calf pods (*n* = 3082), presumably including the majority of singers, were found further offshore with increasing time of the day. Separately, we acoustically localized 217 nearshore singers using vector-sensors. During the day, distances to shore and minimum distances among singers increased, and singers switched more between being stationary and singing while travelling. Together, these findings suggest that the observed diel trends in humpback whale chorusing off Maui represent a pattern of active onshore–offshore movement of singers. We hypothesize that this may result from singers attempting to reduce intraspecific acoustic masking when densities are high nearshore and avoidance of a loud, non-humpback, biological evening chorus offshore, creating a dynamic of movement of singers aimed at increasing the efficiency of their acoustic display.

## 1. Introduction

Animals across many taxa change their activity levels, spatial distribution and behaviour with the time of the day, which may lead to the emergence of small-scale rhythmic, often diel (24 h), spatio-temporal patterns. These can be driven by one or more abiotic and biotic factors including temperature (e.g. [1–4]), light levels (e.g. [5–7]), tide (e.g. [8–11]), food availability and distribution (e.g. [12–15]), predator avoidance (e.g. [16–18]), intra- and inter-specific competition (e.g. [19–21]) and the underlying circadian clock genes (e.g. [22–25]). Diel patterns can be dynamic and may vary with habitat or seasonal changes (e.g. [26–28]). Intraspecific small-scale habitat choices and spatio-temporal patterns may further be influenced by an individual's sex, age and reproductive status (e.g. [18,29–32]). Widespread in the marine environment (e.g. [33–43]), diel migrations often result from a segregation of feeding habitats and resting habitats, although they may also arise from other factors, such as diel changes in the physical environment (e.g. [44]). In this study we investigated small-scale spatio-temporal patterns of habitat use of a migratory, acoustically active cetacean, the humpback whale (*Megaptera novaeangliae*), on its breeding ground.

Humpback whales are migratory capital breeders that occur globally with distinct populations in the different ocean basins [45,46]. In the North Pacific, humpback whales migrate annually between high-latitude summer feeding grounds along the Northern Pacific rim from Russia, through the Aleutian Islands, the Bering Sea, Alaska, Northern British Columbia, to the Northern US West Coast, and low latitude winter breeding areas including Japan, the Philippines, Hawai‘i, Mexico and Central America [47–51]. The Hawaiian archipelago is considered the principal breeding area for the North Pacific metapopulation, with more than 10 000 whales visiting the island chain every year [47,52]. The arrival and departure of humpback whales in Hawai‘i is staggered and segregated by age-class, sex and reproductive condition [53], with peak abundance in the islands typically occurring in February and March [54,55].

Male humpback whales ‘sing’ [56], and this complex acoustic display dominates the local soundscape of many breeding grounds during the winter months [57–61]. In Hawai‘i, changes in ambient sound levels created by numerous males singing concurrently but asynchronously have been linked to changes in overall local whale abundance [62] and these ‘chorusing’ levels display seasonal fluctuations that correspond with the species' migratory patterns [57,60–63]. The current scientific consensus is that song is exclusively produced by males and the timing of singing behaviour is strongly correlated with the breeding season. Song is therefore hypothesized to play an important role in the humpback whale mating system, which is not yet fully understood (see [64] for a comprehensive review on the function(s) of song and discussion of the humpback whale mating system). Most researchers assume song to be associated with inter- and intra-sexual interactions (e.g. [65–67]). The complexity of the sequence of individual units, as well as small variations in the units themselves, in theory provide opportunities for coding of information to communicate fitness to both males and females [68–70].

Throughout their range, humpback whales have been described as a species predominantly found in shallow waters in their summer breeding regions with preferences for nearshore areas and shallow banks [55,71–84]. A correlation between habitat preference and social organization has been shown for humpback whale populations in many breeding areas, including in Ecuador [77,85,86], Madagascar [87], Eastern Australia [80], Peru [88], Puerto Rico [83], Okinawa, Japan [84]) and Hawai‘i (e.g. [55,89]). A breadth of studies specifically examined spatial and temporal habitat use patterns of groups containing a mother–calf pair and showed that females with a calf prefer shallower waters than other pod types (Brazil: [76]; Ecuador: [86]; Madagascar: [87]; Puerto Rico: [83]; Hawai‘i: e.g. [90–97]), a preference that has been proposed as a tactic for avoiding energetically costly and potentially harmful associations with males seeking mating opportunities [90,93].

In contrast to the many studies of habitat use by mother–calf groups on the breeding grounds, relatively few studies have focused on habitat use by singing males. MacKay *et al.* [83] found singers associated with the ledge of the shelf in deep waters further away from shore on a North Atlantic breeding ground off Puerto Rico. In Antongil Bay, Madagascar, singletons (presumed singers) were predominantly found in relatively deeper waters and showed very little variation in occurrence throughout the day [87]. Off the island of Hawai‘i, singing whales were found offshore outside the −200 m isobath as well as in shallower waters, but densities closer to shore were greater than in deeper waters [98]. Individual singers appear to spatially separate themselves [58,98,99] by on average 4 km on a Mexican breeding ground and more than 5 km off Hawai‘i Island, which was significantly greater than the spacing among non-singing lone whales in the same area [98]. The distance of spacing seems to gradually decrease with increasing singer densities [58,64]. Overall, small-scale spatio-temporal patterns of singers and the drivers contributing to these patterns remain largely unknown on many breeding grounds, including in the waters off West Maui, Hawai‘i, which is considered the core habitat within the Hawaiian breeding ground, with highest local whale densities (e.g. [55,71]).

Humpback whale singing activity has been shown to exhibit diel trends with most studies across different breeding grounds reporting an increase in the number of singers or song during the night, for example off Northern Angola [100], Isla Socorro, Mexico [69], Okinawa and Ogasawara, Japan [101,102], as well as at a nearshore recording site off Maui [57]. This nocturnal pattern did not hold for the Abrolhos National Marine Park in Brazil [103]. Additionally, in deeper waters off another Hawaiian island, Kaua‘i, the number of singers and chorusing levels were found to actually increase during the day instead of during the night [63,104]. Hence, diel trends in song occurrence appear to show some variation, with the drivers contributing to these differences in behaviour remaining unclear. Male humpback whales often switch strategies between acoustic display (singing) and engaging in physical competition with other males over access to a female [66,105–108]. Thus, the decrease in singing activity during the day may indicate that daylight and vision play key roles in the formation of competitive groups [57,64] and/or is the result of an increasing group size of whales with progressing time of the day [101,107]. Singers often stop singing when joined by another whale [66,99,108]. Au *et al.* [57] considered an alternative explanation in that the observed diel patterns are the results of singing males moving inshore at night and offshore during the day, but did not discuss possible drivers for such a proposed diurnal migration pattern. These different proposed explanations for the observed diurnal variability in song occurrence have been difficult to resolve without determining the location of singers during different times of the day and night, an effort that has been challenging using acoustic localization, especially in areas such as Hawai‘i with the cacophony of multiple males producing a complex vocalization, and impossible from traditional visual focal-follow studies of singers that are limited to daylight hours.

In this study, we sought to evaluate the hypothesis that humpback whale singers move offshore during the day and closer to shore at night, as well as to identify potential drivers of patterns in singing activity and singer occurrence, by using a combination of acoustic and visual surveys to mitigate the limitations of each individual approach when applied alone. We examined diel patterns of male chorusing from long-term passive acoustic monitoring with multiple shallow- and deep-water recorders off Maui. To resolve the ambiguity of whether observed trends were the results of small-scale migrations of whales or changes in singing activity, we used data from visual land-based surveys to examine patterns in locations relative to spatial features of pods without calves, which are expected to include the majority of singers (e.g. [66,109]). Finally, we used acoustic vector sensors to localize individual singing whales close to shore to understand diel variations in the distance to shore of nearshore singers, their spacing, and their movement behaviour, to help contextualize the visual results and overcome some of the limitations from the land-based visual observations.

## 2. Methods

### 2.1. Data collection

#### 2.1.1. Acoustic monitoring

**Figure 1.**
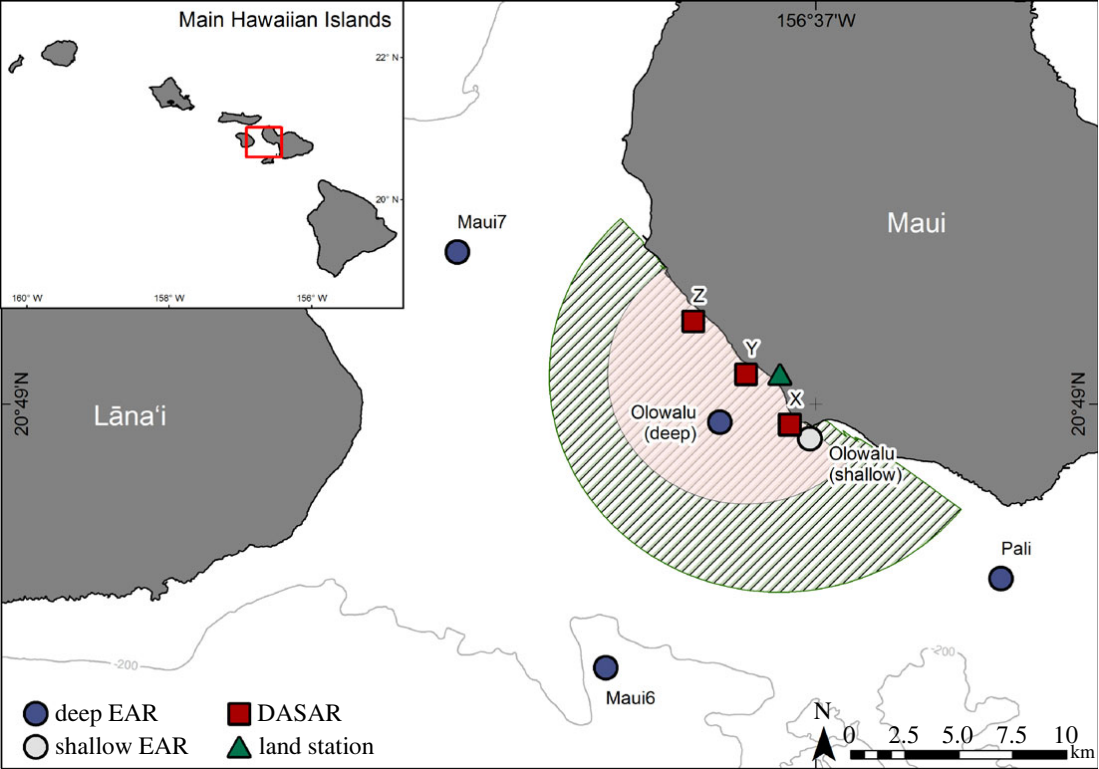
The locations of four deep-water EARs, (dark blue circles), one shallow-water EAR (light-grey circle), and three Directional Autonomous Seafloor Acoustic Recorders (DASARs, red squares) that were deployed for various periods between 2016/17 and 2020/21 off Maui, Hawai‘i. The triangle shows the location of the Olowalu land observation station and the hatched area shows the 10 km field of view. The red shaded area indicates the 6 km radius from DASAR Y. Inset shows the study area within the Main Hawaiian Islands.

Acaoustic data were collected with four deep-water and one shallow-water bottom-moored autonomous Ecological Acoustic Recorders (EARs) using a Sensor Technology SQ26–01 hydrophone with a sensitivity of −193.5 dB re 1 V µPa^−1^ [110] during the humpback whale breeding seasons (*ca* December to April) of 2016 to 2021 at five locations off Maui, Hawai‘i (figure 1). Deployment locations, depths, recording periods and sampling information are summarized in table 1. All sites were within or near the −200 m isobath, a depth within which most humpback whales occur in Hawai‘i (e.g. [55,71,98]) and located on relatively flat bathymetry (deep EARs) or near a coral reef (shallow EAR). The diver-deployed shallow EAR was anchored to a concrete block on the bottom; the deep EARs were deployed off a small vessel and were each coupled with a syntactic foam float, two SubSea Sonics AR-60 acoustic releases or one EdgeTec PORT LF Push Off acoustic release, and approximately 75 kg of sandbags to anchor the mooring. These moorings positioned the hydrophone approximately 4 m off the bottom in the water column. EARs recorded at a sample rate of either 25 or 50 kHz (table 1) and a 10% duty cycle, recording for 30 s every 5 min. Acoustic data were analysed for each survey year from 1 December to 30 April.

**Table 1.** Summary of passive acoustic monitoring sites, location, depth and distance to shore for continuous recording periods (as mm/dd/yy) during the Hawaiian whale season (approx. December through April) 2016/17 through 2020/21 for four deep and one shallow EARs and three Directional Autonomous Seafloor Acoustic Recorders (DASARs) deployed off Maui, Hawai‘i.

site	recorder type	lat (°N)	long (°W)	depth (m)	distance to shore (m)	duty cycle (s)	sample rate (kHz)	recording periods (mm/dd/yy)
Olowalu (shallow)	EAR	20.802	−156.619	10	547	30/300	50 50 50 25 25 25	07/30/16–01/08/17; 01/09/17–05/17/17; 12/07/17–05/17/18; 11/02/18–03/13/19; 11/22/19–05/08/20; 12/11/20–06/19/21
Maui 7	EAR	20.880	−156.766	42	6748	30/300	50	01/04/17–05/04/17
Olowalu (deep)	EAR	20.809	−156.656	62	2939	30/300	50 50 25 25	12/22/16–05/04/17; 12/01/18–04/25/19; 11/06/19–03/29/20; 12/01/20–05/26/21
Pali	EAR	20.744	−156.539	85	3460	30/300	50	12/22/16–05/04/17
Maui6	EAR	20.707	−156.704	258	13 967	30/300	50 50 50 25 25	12/22/16–05/04/17; 12/07/17–04/30/18; 12/01/18–04/24/19; 11/15/19–05/15/20; 12/07/20–05/26/21
X	DASAR	20.808	−156.627	19	350	continuous	1	03/11/20–07/01/20
Y	DASAR	20.829	−156.646	18	499	continuous	1	03/11/20–07/01/20
Z	DASAR	20.851	−156.668	20	507	continuous	1	03/11/20–07/01/20

Vector sensor acoustic data were collected in shallow waters off West Maui with three Directional Autonomous Seafloor Acoustic Recorders (DASARs) between 11 March 2020 and 1 July 2020 (figure 1 and table 1). Autonomous DASAR packages are equipped with an omnidirectional acoustic pressure sensor with a sensitivity of −149 dB re 1 V µPa^−1^ at 100 Hz and two-dimensional particle velocity sensors [111,112]. The three DASARs were spaced in-line approximately 3 km apart at approximately −20 m depth. The instruments were lowered to the bottom with a rope from a small vessel and the orientations for each DASAR were acoustically calibrated as described in Thode *et al.* [113] and Tenorio-Halle *et al.* [114]. DASARs recorded continuously with a 1 kHz sample rate. Acoustic data were analysed for the period between 1 and 30 April 2020. These data were collected as part of a separate project, dictating deployment and recovery schedules, instrument settings and necessitating a trade-off between different research questions and analyst availability, which limited the total number of analysed days with larger numbers of singers.

#### 2.1.2. Land-based visual observations

Visual land-based scan samples [115] were conducted one to three times each week throughout each whale season from a hill near Olowalu on West Maui (20.829° N, −156.6315° W, 83.7 m elevation; figure 1). The total field of view was slowly scanned for whales with a pair of Bushnell 7 × 50 marine binoculars from one end across to the other once per scan by the same experienced observer during all surveys. Scans took place for 30 min every hour, up to seven times starting at 8.30 local standard time during the first 2 years and at 8.00 local standard time during the subsequent 2 years, weather permitting. The scanning direction was alternated during the first year and randomized during all subsequent years to reduce bias by inadvertently spending unequal amounts of time surveying an area segment at the beginning or the end of each scan. Any humpback whale or group of humpback whales sighted by the observer, regardless of number of individuals, was called a pod. When a pod was sighted, the location in vertical and horizontal angles was fixed by the observer with a Lietz DT5 digital surveyor's theodolite connected to a computer and digitally transferred upon prompt by a computer operator while the observer continued their scan. The geo-referenced program Pythagoras [116] automatically converted the time-stamped angles into latitude and longitude coordinates in real time and calculated the distance of the fix from the land station. Additional metadata recorded for each fix included the observable pod composition and group size (1, 2 or ≥3 whales) as called out by the observer. For the purpose of this paper, we only differentiated between pods containing a calf (typically either a lone mother–calf pair or a mother–calf pair escorted by one or more males) and any pod type without a calf *(non-calf pod*). A detailed discussion on definitions of group compositions during land-based surveys can be found in Kügler *et al.* [62]. Because pod sizes and compositions were immediate assessments and no prolonged time was spent observing pods before continuing with a scan, these were considered conservative estimates, with the presence of a calf probably under-reported because of its relatively small size and profile at the surface.

If a pod could not be fixed with the theodolite, compass bearing and distance in reticles were determined with the binoculars and noted as a time-stamped ‘non-fix’ with the same composition and behaviour information as fixes. During each scan, no pod was recorded twice to avoid pseudo-replication. Scans were conducted in a non-focal-follow manner and regarded as discrete; thus any pod was recorded as a new pod in subsequent scans, even if previously fixed. Environmental data (glare, visibility/haze, Beaufort sea state, wind speed, wind direction) were collected prior to each scan and when conditions changed during a scan. Scans were conducted in conditions of Beaufort sea states of 3 or less (to maximize the sightability of whales; cf. methods described in e.g. [93]), wind speeds at the station under 15 knots and dry conditions. The land station is exposed to strong onshore winds typically occurring in the afternoons, as well as limited access to the site that was only accessible through a recycling centre that closed at 14.30 local time, both precluding observations in the late afternoon hours. Only survey days with two or more completed scans were included in the analysis.

### 2.2. Data processing and analysis

#### 2.2.1. Acoustic EAR data

All acoustic EAR recordings were down-sampled to a 3 kHz sample rate using Matlab (Matlab and Statistics Toolbox Release 2018b, The MathWorks, Inc., Natick, MA, USA), resulting in a 0–1.5 kHz analysis bandwidth, which corresponds to the range where the majority of humpback whale song units have their peak frequencies [56,117] (figure 3; also see electronic supplementary material, figure S1). A custom Matlab script converted each original and down-sampled 30 s recording from 16 bit sample values into volts and then calculated the root-mean-square sound pressure levels (RMS SPL) in dB re 1 μPa for the full frequency band (0–12.5 kHz or 0–50 kHz, *‘fullband’*), 1-Octave frequency bands and the down-sampled 0–1.5 kHz samples, as

**Figure 2.**
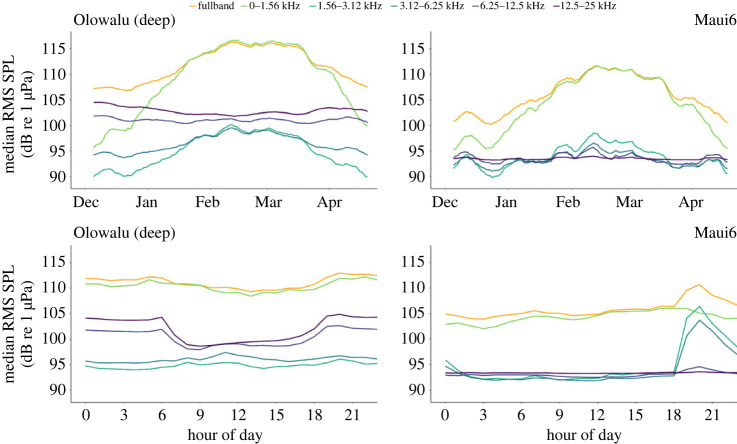
Seasonal and diel patterns of sound levels (RMS SPL, in dB re 1 μPa) recorded between 1 December 2018 and 25 April 2019 at the nearshore Olowalu (deep) and offshore Maui6 EAR sites off Maui, Hawai'i, in the 0–25 kHz (*fullband*) and 1-Octave frequency bands. Daily median RMS SPLs (top panels) were smoothed with a running average of *n* = 10.

**Figure 3.**
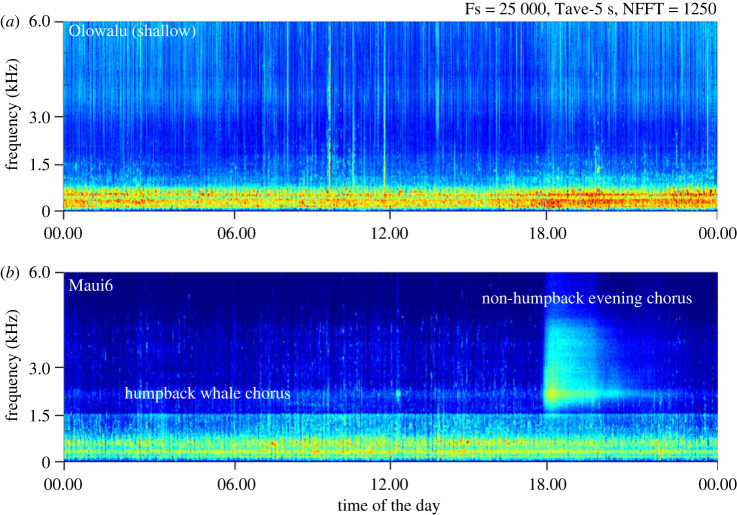
Long-time spectral average spectrograms between 0 and 6 kHz of 24 h recorded at (*a*) the nearshore Olowalu (shallow) site and (*b*) the offshore Maui6 EAR site during the 2019/2020 whale season, illustrating the average daily marine soundscape off Maui, Hawai‘i during the peak of the humpback whale season in February. Yellow and red colour hues indicate higher levels of acoustic energy.

2.1 $$\mathrm{RMS}\;\mathrm{SPL}=20\log\sqrt{\frac{1}{T}\overset{T}{\underset{0}{\int }}p^{2}(t)dt}$$

where *T* is the duration of each file (30 s) and *p(t)* is the pressure *p* re 1 μPa at time *t*. Humpback whale chorusing is the dominant source of low-frequency acoustic energy during the whale season (figure 2), therefore RMS SPL is a representative metric of the cumulative amount of singing during any given 30 s recording period [57]. In addition, the 1.56–3.12 kHz band was selected for analysis of the ambient sound not associated with song production, to investigate possible noise impacts on humpback whale chorusing (figures 2 and 3 and electronic supplementary material, figure S1).

All subsequent data processing and statistical analyses were conducted in R 4.0.1 [118]. The median RMS SPL in dB re 1 μPa for the 0–1.5 kHz and 1.56–3.12 kHz frequency bands were calculated for each hour of each day and recording site. The use of the median reduces auto-correlation within the time series, since recordings are not independent from each other whenever a humpback whale's song or environmental noise is continuous over multiple recordings. It also reduces the impact of outliers from intermittent high-intensity noise (such as transiting small vessels) and from individual whales singing close to the recorders, temporarily increasing SPL levels. For both frequency bands, the minimum, maximum and maximal difference of hourly median RMS SPL levels in dB re 1 μPa (ΔdB = median(RMS SPL)_min_ − median(RMS SPL)_max_) were also calculated for each month and across the entire season.

#### 2.2.2. Land-based visual observations

Compass bearings and reticle distances of all non-fix observations from the land station were converted into latitude and longitude coordinates with an adapted version of the *whalemap()* function of the *bangarang* package [119]; the function also calculates distance to the observer, which is the equivalent of distance to the shore station calculated for fixes by Pythagoras. All pods with distances greater than 10 km were excluded from analysis (figure 1). This cut-off was chosen as a compromise between proximity to the offshore EAR recorder location and the declining detection probability, especially of calves, with increasing distance from the land observation station. Because unambiguous identification of a singer is challenging using visual shore-based observations alone, we used non-calf pods as a proxy for singers. While most singers are typically lone individuals, not all lone individuals are males or sing [109,120]. Singers can also be a member of a dyad or larger group [108,121]. By contrast, most singers are found in pods without calves (e.g. see [66,109]). Thus, focusing on non-calf pods accurately reflects the predominant occurrence of singers.

Bathymetry data for the area were obtained from the Main Hawaiian Islands Multibeam Bathymetry Synthesis project (http://www.soest.hawaii.edu/hmrg/multibeam/bathymetry.php) as a 5 m grid and converted to text file in ArcGIS Desktop/ArcMap 10.8.1 (Environmental Systems Research Institute, Redlands, CA). The raster text file was then read into R with the *brick()* function from the *raster* package [122], converted into a dataframe with columns for latitude, longitude and depth, saved as a csv file, and read in as proprietarily formatted bathymetry data with the *read.bathy()* function of the *marmap* package [123]. For all whale pod positions in latitude and longitude, depth was determined with the *get.depth()* function, and distance to shore with the *dist2isobath()* function with isobath set to 0, all from the *marmap* package. Summary statistics for the two metrics (depth, distance to shore) were calculated for the entire dataset as well as per year.

All time-stamped observations were rounded to the full hour with scans conducted between *.00 and *.30 (first 2 years) floored to the hour and scans conducted between *.30 and *.00 (last 2 years) rounded up. Medians per hour for the number of observed pods, distance to shore and depth were calculated and pooled for the entire dataset and per year. Differences between the medians of the earliest scan hour of the day and the last scan hour of the day were also calculated.

#### 2.2.3. Acoustic DASAR data

**Figure 4.**
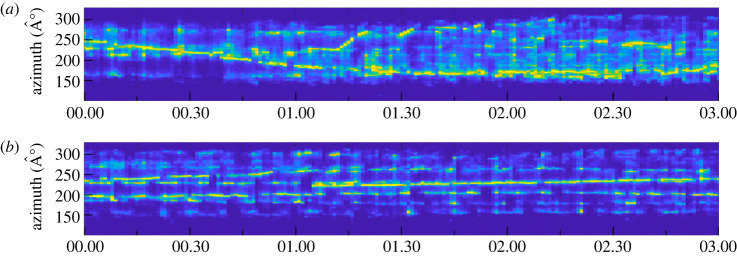
Examples of AHDs with a 0.75 NTV filter of 3 hours during (*a*) high humpback whale chorusing activity on 4 April 2020, and (*b*) low chorusing activity on 14 April 2020. Continuous yellow tracks represent songs from individual singers received at the DASAR from different directions (azimuth).

Azigrams, time-frequency visualizations of the dominant directionality of ambient sound [124], were calculated for 24 h periods for each DASAR with a custom Matlab algorithm. Azimuthal histogram displays (AHDs) then stacked histograms of azimuths from discrete sound sources binned over 60 s to generate a single image that summarizes azimuthal information over long time scales [114]. A metric called normalized transport velocity (NTV) was used to reduce contributions from diffuse background noise from the images. As a result of this processing, continuously singing individual humpback whales appear as azimuthal tracks over time (figure 4). See Tenorio-Halle *et al.* [114] for specifics on this process.

Using AHDs over 3 h windows for 24 h samples from midnight to midnight local standard time, all visible tracks were manually traced by trained analysts across all three DASARs using a custom Matlab script [114]. The script then extracts all azimuthal tracks and matches them between DASARs. Every 60 s the script calculates a ‘score’ metric for every possible cross-track pairing between the DASARs. A high score implies a higher likelihood that two azimuthal tracks between two DASARs arise from the same singer. For each cross-track pairing, median scores were calculated and displayed as confusion matrices. High median scores indicated correct matches of the same singer between DASARs. Successfully matched azimuthal tracks were then triangulated using the three azimuths from the three DASARs and the positions in latitude and longitude of individual singers were localized every 60 s. For this study, only tracks that appeared on all three DASARs were localized, and thus durations of localized tracks were limited by the shortest track on any of the three sensors. Tracing was done conservatively; unless they could be unambiguously separated, azimuthal tracks were traced as separate events if two or more tracks crossed or individual tracks showed longer than 5 min.

All localizations were displayed on two-dimensional maps of the region and visually investigated for unrealistic localizations, which were then removed if necessary. To avoid pseudo-replication, tracks that had been originally traced as separate individual tracks were subsequently combined by adjusting track IDs, if the resulting two-dimensional trajectories indicated that they belonged to the same whale. AHDs displaying the labelled azimuthal tracks were cross-referenced to ascertain that those tracks did not occur concurrently, which would have indicated that tracks belong to two whales singing in close proximity.

To identify individual localizations within tracks that were outliers, the closest distance between localizations were calculated using the *dist()* function from the *stats* package [118] for each track. A localization was considered an outlier and removed if the closest distance was outside the 95% quantile of all within-track distances. To reduce localization inaccuracy, coordinates of each track were corrected with a running average over five consecutive time steps in the *rollapply()* function from the *zoo* package [125].

Tracks of distant singers are difficult to identify on AHDs during high chorusing activity and are not localizable (figure 4). Therefore, because the lack of localized offshore singers cannot be used to infer absence of singers during those times, only singers localized within a 6 km radius of DASAR Y were included in the subsequent analysis. Based on the distribution of distances from DASAR Y (electronic supplementary material, figure S2) a cut-off range of 6 km was chosen as a distance within which detectability of singers is high. To reduce possible non-independence of samples within the continuous data, only localizations within the first 30 min of every hour were considered. The duration of the sampling window was chosen to allow for gaps in localizations when whales were surfacing (and therefore not singing) and masking of singers from transient environmental noises such as vessels, which intermittently precluded localization. From these subsampled data, the average location per hour was calculated for all singers by computing the mean of all coordinates for the individual tracks. From these, distances to shore where then determined with the *dist2isobath()* function with isobath set to 0 from the *marmap* package, and distances to the respective nearest neighbours were calculated using the *dist()* function from the *stats* package. Hourly distances to shore and nearest neighbour distances were averaged across all days.

For each localized singer, the state was determined as either ‘*stationary*’ or ‘*travelling*’ for every 60 s localization, based on the whale's movement speed as calculated from the distance between subsequent localizations. Distances used to compute speed were calculated from the smoothed tracks assuming Euclidean geometry. If the speed exceeded 2 km h^−1^, the singer was considered ‘*travelling*’ (after [98,114]), and if it was below this value, the sample was assigned ‘*stationary*’ state. Time-stamps of localizations were rounded downward to the hour and for each whale the percentage of time spent travelling versus remaining stationary per hour was determined. If the singer spent less than 70% of its time in either of the two states, it was assigned a ‘*both*’ state for this hour. Finally, for every hour, the percentage of singers in each of the three states (stationary, travelling, or both) was calculated.

### 2.3. Statistical analysis

We tested the hypothesis that, during the day, singers tend to move offshore instead of decreasing singing activity, using three different datasets of ambient sound measurements (EARs), visual observations, and singer localization and tracking (DASARs).

A series of multivariate analyses were conducted by fitting generalized additive models (GAMs) to the data to investigate small-scale temporal patterns of sound levels, visual whale sightings in relation to habitat variables, and presence and behaviour of singers. To control for variability that results from the whales' natural migratory pattern as well as interannual fluctuations, variables for seasonality and survey year were included in all models. For the models examining sound levels across multiple EARs, covariates for the recorder location were also included as a blocking variable. Combined, the model results from these three different datasets provided complementary information towards an overall picture of singer occurrence and behaviour off Maui and helped answer our hypothesis.

All GAMs were fit with the *mgcv* package [126] in R. All significances for smoothing and parametric terms were determined with the *anova.gam()* function from the *mgcv* package using Wald tests with *α* = 0.05. The residuals of all models were checked for autocorrelation with the *acf()* function from the *stats* package.

#### 2.3.1. Acoustic EAR data

Variability in sound levels (RMS SPL) across shallow and deep locations in the humpback whale frequency band (0–1.5 kHz) and the frequency band corresponding to non-humpback whale environmental sounds (1.56–3.12 kHz) was each fit with the following equation:

2.2 $$\mathrm{RMS}\;\mathrm{SPL}=\beta_{0}+f_{1}(\mathrm{day}.\mathrm{season})+f_{2}(\mathrm{hour})\cdot \mathrm{site}+\mathrm{site}+\mathrm{year}+\epsilon$$

where *β_0_* is the intercept, *f_1_* and *f_2_* are the respective smoothing functions, *day.season* is the count of days into the season of each date, with the first day of the season defined as 1 December, *hour* is the hour of the day as a cyclic spline, *site* is a factor for the five EAR locations, *year* is a factor for the year corresponding with the peak for each of the five whale seasons, and *ε* is an error term. While interaction terms for *f*(*day.season hour) site* in the full models were significant for both frequency bands, visual investigation of effect plots showed no strong patterns in the effect heatmaps. Because additional diel effects remained even when including the interaction term, we decided to fit the model without the interaction to reduce the number of model terms for better statistical power and to avoid overfitting. The residuals of the GAMs showed considerable autocorrelation. We therefore refit the models for both frequency bands as generalized additive mixed models (GAMM) with the *gamm()* function from the *mgcv* package by including an additional correlation structure as *correlation* *=* *corAR1(form* *=* *∼ 1|Date+site)* that mostly reduced any considerable autocorrelation in the data.

To further investigate these changes in diel patterns across a season, an additional GAMM was fit for a subset of the data corresponding with the beginning (December), peak (February) and end (April) of the whale season for each frequency band as

2.3 $$\mathrm{RMS}\;\mathrm{SPL}=\beta_{0}+f_{1}(\mathrm{day}.\mathrm{season})+f_{2}(\mathrm{hour})\cdot \mathrm{site}\cdot \mathrm{time}.\mathrm{season}+\mathrm{site}+\mathrm{year}+\epsilon$$

where *time.season* is a categorical variable for the three discrete segments of the season (early, peak, late), which reflect times of lowest and highest whale densities. Because little to no data were available during December for the Maui7 and Pali sites, these sites were excluded from this second analysis.

#### 2.3.2. Land-based visual observations

Two separate GAMs were fit to the land-based data for non-calf pods to investigate temporal patterns of whale occurrence in relation to environmental parameters that would indicate a shift in distribution within the survey area, using the following equations:

2.4 $$d_{\mathrm{shore}}=\beta_{0}+f_{1}(\mathrm{day}.\mathrm{season})+f_{2}(\mathrm{hour})\cdot \mathrm{pod}.n+\mathrm{pod}.n+\mathrm{year}+\epsilon$$

2.5 $$\mathrm{depth}=\beta_{0}+f_{1}(\mathrm{day}.\mathrm{season})+f_{2}(\mathrm{hour})\cdot \mathrm{pod}.n+\mathrm{pod}.n+\mathrm{year}+\epsilon$$

where *d*_shore_ is the distance to shore, *depth* is the depth, and *pod.n* is a factor for the number of whales per pod (1, 2, ≥ 3 non-calf animals). Interaction terms for the smoothers of day of the season and hour were not significant and were therefore not included in the final models.

#### 2.3.3. Acoustic DASAR data

To determine whether nearshore singers change their presence and behaviour with time of the day, the following GAMs were fit:

2.6 $$d_{\mathrm{shore}}=\beta_{0}+f_{1}(\mathrm{day}.\mathrm{season})+f_{2}(\mathrm{hour})+f_{3}(d_{Y})+\epsilon$$

2.7 $$d_{\min,\mathrm{neighbour}}=\beta_{0}+f_{1}(\mathrm{day}.\mathrm{season})+f_{2}(\mathrm{hour})+f_{3}(d_{Y})+\epsilon$$

2.8 $$p.\mathrm{behaviou}r_{\mathrm{state}}=\beta_{0}+f_{1}(\mathrm{day}.\mathrm{season})\cdot \mathrm{behaviou}r_{\mathrm{state}}+f_{2}(\mathrm{hour})\cdot \mathrm{behaviou}r_{\mathrm{state}}+f_{3}(d_{Y})\cdot \mathrm{behaviou}r_{\mathrm{state}}+\epsilon$$

where *d*_shore_ is the minimum distance to shore of individual singers within 6 km of DASAR Y and *d*_min,neighbour_ is the distance to the nearest singing neighbour. For diel patterns of behaviour, a binomial GAM was fit, where *p.behaviour* is the probability of a whale spending at least 70% of its track during a given hour in one of three *states*: remaining stationary, travelling, or both. Behaviour states were assigned binary 0 or 1 values for each of the three states for each individual singer per hour. To control for potentially changing detection probability with distance to the recorders and/or unequal distribution of singers in the survey area (electronic supplementary material, figure S2), distance to DASAR Y (d_Y_) was included in the models as the median distance per track per hour.

## 3. Results

**Figure 5.**
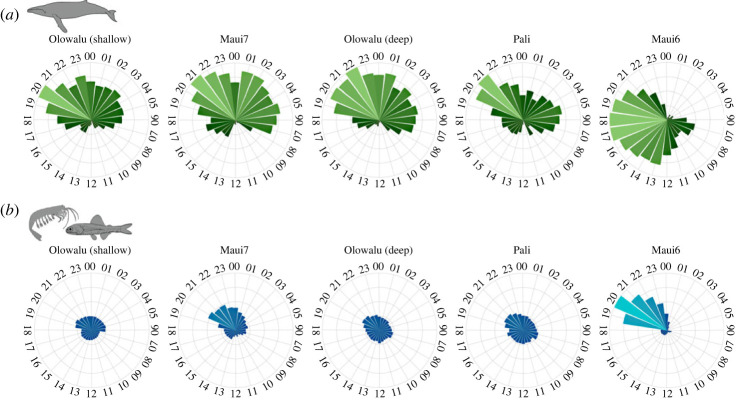
Diel patterns of standardized sound levels (median hourly RMS SPL in dB re 1 μPa) in two frequency ranges off Maui, Hawai‘i across five EAR recording sites. (*a*) Levels in the 0–1.5 kHz humpback whale frequency band. (*b*) Levels in the 1.56–3.12 kHz frequency band associated with non-humpback, biotic environmental sounds (diffuse ambient noise from e.g. snapping shrimps, fish chorusing). For visualization of patterns, levels were normalized by the overall median per site per year, and medians per hour were then standardized between 0 and 1 within sites for (*a*) and across sites for (*b*) due to differing magnitudes of average daily changes among sites and frequency bands. Sites are ordered by depth from shallow to deep.

**Figure 6.**
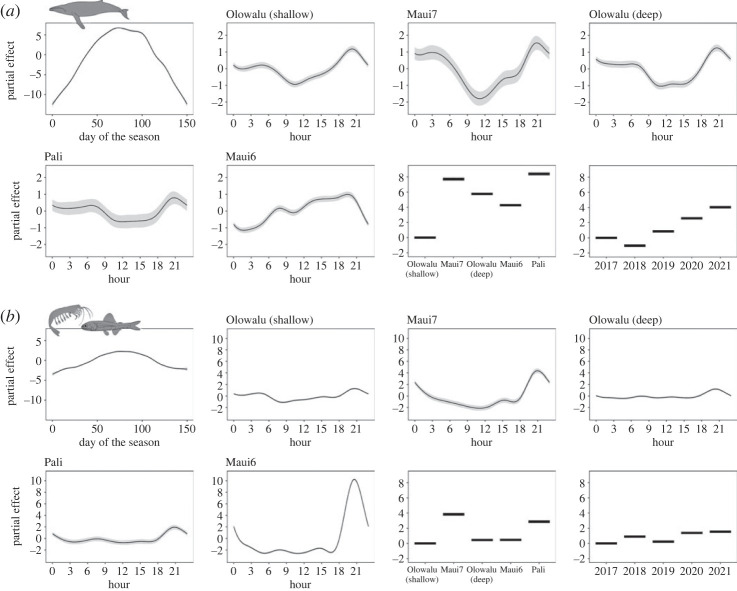
Patterns of sound levels (hourly median RMS SPL in dB re 1 μPa) recorded at five EAR locations off Maui, Hawai‘i in (*a*) the 0–1.5 kHz humpback whale frequency band and (*b*) the 1.56–3.12 kHz frequency band associated with non-humpback, biotic environmental sounds (diffuse ambient noise from e.g. snapping shrimps, fish chorusing). Plots show the partial effects from GAMMs for the explanatory variables day of the season (where day_1_ = 1 December), hour of the day by recording site in local standard time, and the covariates recording site and the year corresponding with the peak of each whale season. Shaded areas indicate confidence intervals.

EAR recordings during the humpback whale breeding season were obtained for four to five consecutive years between 2016 and 2021 for three recording sites and one season (2017) at the Maui7 and Pali sites (table 1 and figure 1). A summary of descriptive statistics for sound levels can be found in electronic supplementary material, table S1. RMS SPL hourly medians in the 1.56–3.12 kHz frequency band, on average, were lower and showed fewer variations than levels in the 0–1.5 kHz band at all sites. In addition to seasonal patterns and interannual and site-specific variability, pronounced diel patterns in the 0–1.5 kHz frequency band were found at all sites, but trends were less distinct and more varied among sites in the 1.56–3.12 kHz band, with all sites showing a sunset peak of varying magnitude (figures 5 and 6).

A total of 71 visual surveys from the Olowalu land station consisting of at least two scans were completed (2017: *n* = 19, 2018: *n* = 25, 2019: *n* = 16, 2020: *n* = 10; electronic supplementary material, table S2), with some variability in total effort per scan hour within and across all three years (electronic supplementary material, table S2). During these surveys, a total of 3082 non-calf pods were recorded (2017: *n* = 749, 2018: *n* = 943, 2019: *n* = 722, 2020: *n* = 668; electronic supplementary material, table S3), which showed varying patterns of seasonality and time of the day, but not across years, for the examined habitat variables.

Eight days of DASAR data between 1 and 29 April 2020 were analysed, resulting in a total of 340 individually localized singers, of which 217 were within 6 km of DASAR Y (electronic supplementary material, table S4). Nearshore singers showed variability in numbers, spacing and movement behaviour across the survey month as well as the diel scale.

### 3.1. Impacts of seasonality on patterns in acoustics and whale occurrence

**Figure 7.**
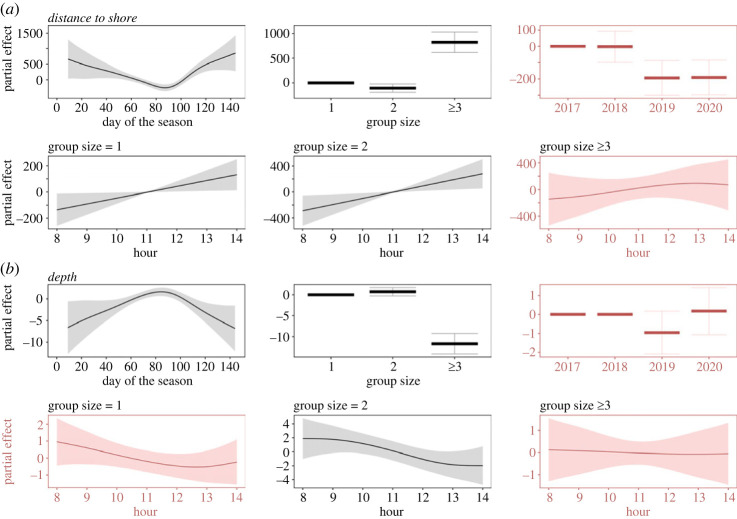
Spatial and temporal patterns of occurrence of non-calf humpback whale pods recorded during visual observations from the Olowalu land station on Maui, Hawai‘i. Plots show the partial effects of variables fit with GAMs for (*a*) distance to shore (in metres), (*b*) depth (in metres), and the explanatory variables day of the season (where day_1_ = 1 December), hour of the day between 8.00 and 14.00 local standard time by group size (1, 2, ≥3 non-calf whales), and the covariates for group size and the year corresponding with the peak of each whale season. Shaded areas and error bars indicate confidence intervals. Red colours indicate predictors were not significant.

**Figure 8.**
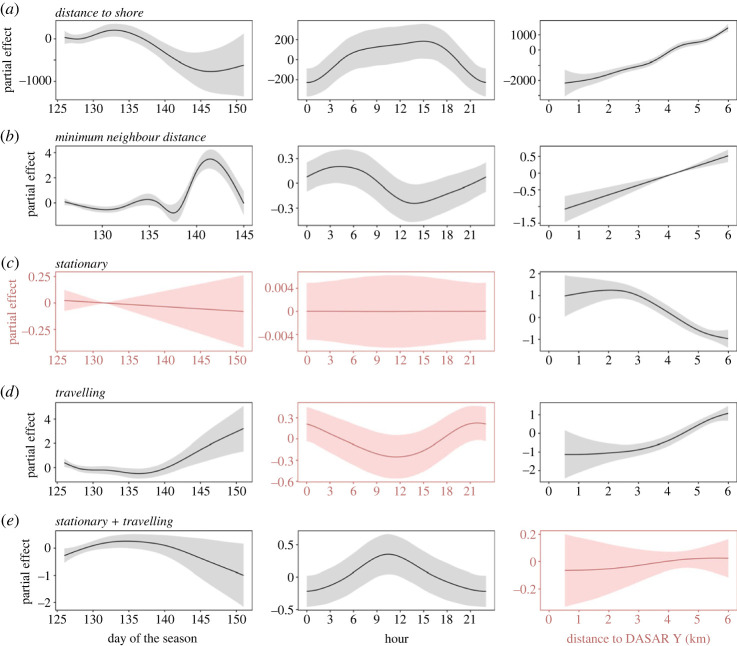
Seasonal and diel patterns of occurrence and behaviour of singers off Maui, Hawai‘i in April 2020 that were acoustically localized during 30 min every hour from vector sensor (DASARs) recordings. Plots show the partial effects of explanatory variables fit with GAMs for (*a*) distance to shore of mean position of singers (in metres), (*b*) minimum distance to the nearest neighbour of mean positions of singers (in kilometres), (*c*) the probability per hour that a singer remained stationary, (*d*) the probability per hour that a singer was travelling, (*e*) the probability per hour that a singer was both travelling and stationary, and the explanatory variables day of the season (where day_1_ = 1 December), hour of the day in local standard time, and distance to DASAR Y. Shaded areas indicate confidence intervals. Red colours indicate predictors were not significant. Only singers within 6 km of DASAR Y were included in the models.

Seasonal changes in RMS SPL levels obtained from five EAR recording sites contributed significantly to variability in ambient sound between December and March in both frequency bands (figure 6 and electronic supplementary material, table S5), with levels in the 0–1.5 kHz humpback whale band changing on average by over 10 to 15 dB at all sites between the start and tail ends of the humpback whale season and the peak of the season in February (figure 6*a*). The overall seasonal change in levels in the 1.56–3.12 kHz frequency band was comparatively smaller at approximately 4 dB (figure 6*b*) and probably attributable to some influence from humpback whale song at higher frequencies (figure 2). The occurrence of visually observed non-calf pods in relation to distance to shore and depth significantly varied across the whale season, but not among years (figure 7 and electronic supplementary material, table S5). However, sample sizes for the beginning and end of the whale seasons were small (electronic supplementary material, table S2), and interpretations need to be inferred with caution. Further, individual singers acoustically localized during the month of April 2020, which reflects the tail end of the humpback whale season off Maui, showed significant changes on a daily scale across the survey month (figure 8 and electronic supplementary material, table S5). The DASAR data revealed that the average distance to shore of singers decreased throughout the month and they were increasingly spaced further apart. Whales were also moving more often while singing late in the season.

### 3.2. Diel humpback whale chorusing and ambient sound patterns

All EAR recording sites showed significant diel patterns in the 0–1.5 kHz humpback whale frequency band (electronic supplementary material, table S5). RMS SPLs at the deep offshore EAR (Maui6) tended to gradually increase from morning through the day, by on average 3.2 dB, and maintain high levels into the late afternoon before rapidly decreasing after approximately 19.00–20.00 (figure 6*a*, electronic supplementary material, table S6). By contrast, levels tended to decrease during the day and remained relatively stable during the night-time in the nearshore area (figure 6*a*). At the nearshore Olowalu (shallow), Olowalu (deep) and Pali sites, as well as the mid-channel Maui7 site, RMS SPL levels changed by on average 2.8 (Pali) to 4.4 (Maui7) dB over the course of the day (electronic supplementary material, table S6).

All sites also showed significant diel patterns in the 1.56–3.12 kHz frequency band (electronic supplementary material, table S5). All nearshore sites and the mid-channel Maui7 site showed no strong changes in levels during most of the day with a narrow peak at approximately 18.00–19.00 local time, temporarily increasing baseline levels by approximately 2.5–2.6 dB at the two Olowalu and the Pali sites and by 7.3 dB at the Maui7 site (figure 6*b* and electronic supplementary material, table S6). At the deep offshore Maui6 site, a peak occurred around the same time as the other sites, but levels increased by over 15 dB, raising the noise level of the ambient sound in that frequency band almost 30-fold. This difference in magnitude of the evening peak is also visualized in figure 5. All sites except Maui7 showed an additional smaller peak of temporarily increased levels around sunrise (*ca* 6.00–7.00 local time) of less than 1 dB (figure 6*b*).

Diel patterns in the 0–1.5 kHz humpback whale frequency band differed markedly during the beginning, peak and end of the season (electronic supplementary material, figure S3*a*). The pronounced daytime decrease in nearshore waters appears to only be prominent during the peak of the season; at the beginning and end of the season, levels gradually increased throughout the day before decreasing around sunset. By contrast, diel patterns in the 1.56–3.12 kHz band remain fairly consistent during these three times of the whale season both at the shallow nearshore/mid-channel and the deep offshore sites (electronic supplementary material, figure S3*b*), with a slight shift in timing of the morning and evening peaks that corresponds with changing sunrise and sunset times between December and April. Relating the diel patterns of both frequency bands (electronic supplementary material, figure S3*b*) reveals that during the peak of the humpback whale season at the offshore site, levels in the 0–1.5 kHz band start decreasing at approximately 18.00 local time at the same time levels in the 1.56–3.12 kHz band begin to rise, while the evening peak in both frequency bands occurs at the same time early and late in the season. At the shallow Olowalu site, morning and evening peaks in both frequency bands occur at the same time during the peak and tail end of the whale season, but are slightly shifted at the beginning of the season. These results also indicate that the sources of the dominant acoustic energy in the two frequency bands are not completely discrete to these frequencies, but have some frequency range overlap that impacts the respective other frequency band during periods of high acoustic activity (figure 2).

### 3.3. Diurnal patterns of non-calf pod occurrence from visual surveys

Non-calf pods consisting of one or two whales appeared to move away from shore into deeper waters between morning and early afternoon hours, with distance to shore increasing (one whale: ed.f. = 0.836, *F* = 0.814, *p* = 0.0153; dyad: ed.f. = 0.877, *F* = 1.029, *p* = 0.0076; figure 7*a* and electronic supplementary material, table S5) by an average of between 81 m (2019) and 772 m (2017) (electronic supplementary material, table S7), and depth decreasing (figure 7*b*) with increasing time of the day. However, depth only seemed to change marginally by less than 4 m (figure 7*b* and electronic supplementary material, table S7) and this trend was only significant for dyads (ed.f. = 1.206, *F* = 0.651, *p* = 0.0372), but not for pods with one animal (ed.f. = 0.99, *F* = 0.329, *p* = 0.1307). Pods with three or more whales were already found in deeper waters further away from shore compared with the pod types with fewer whales and did not show any significant diurnal patterns (figure 7 and electronic supplementary material, table S5). Visualizations of all partial effects of the GAMs are summarized in figure 7.

### 3.4. Diel patterns of individual singers

Acoustically localized individual singers showed variation in both occurrence and behaviour between different times of the day. The distance to shore increased during the day and decreased at night (ed.f. = 2.706, *F* = 1.419, *p* = 0.033; figure 8*a* and electronic supplementary material, table S5), with median distances to shore ranging from 2.3 to 4.4 km (electronic supplementary material, table S8). Median distances among singers were greatest in the morning (median (IQR): 3.33 (1.27) km) and decreased to less than 1 km (median (IQR): 0.73 (1.46) km) in the afternoon and again at approximately 21.00 (median (IQR): 0.96 (2.90) km) (electronic supplementary material, table S8) and this trend of morning dispersal and early afternoon clustering was significant (ed.f. = 1.998, *F* = 0.786, *p* = 0.0241; figure 8*b* and electronic supplementary material, table S5). No significant relationship was found between the probability of singers to remain stationary or to travel and time of the day (figure 8*c*,*d*); these behavioural states were largely determined by distance to DASAR Y (electronic supplementary material, table S5), with the probability for singers to remain stationary decreasing (ed.f. = 2.495, *χ*^2^ = 62.46, *p* ≪ 0.001) and the probability of travelling increasing (ed.f. = 2.344, *χ*^2^ = 50.07, *p* ≪ 0.001) with increasing distance to DASAR Y. However, although not significant, nearshore singers appeared to become less likely to travel during midday, but switched more frequently between being stationary and travelling (travel: ed.f. = 1.549, *χ*^2^ = 3.788, *p* = 0.064; both: ed.f. = 1.867, *χ*^2^ = 5.541, *p* = 0.0287; figure 8*d*,*e* and electronic supplementary material, table S5).

## 4. Discussion

Male humpback whale song dominates the soundscape of many breeding grounds during the winter and spring months [57–60] and is hypothesized to play an important role in their mating system [64,127]. Nevertheless, small-scale spatio-temporal patterns of habitat use by singers remained poorly understood with conflicting trends reported among different breeding grounds as well as within the Hawaiian breeding ground [57,63,69,87,100,101,104]. Our findings indicate that the observed diel chorusing patterns off Maui are caused by several factors and are in part the result of singers moving offshore during the day and closer to shore at night, rather than exclusively a daytime shift in dominant behaviour from singing to non-singing activities.

### 4.1. Humpback whale chorusing

Humpback whale chorusing levels recorded at all sites were predominantly impacted by seasonal changes in local whale abundance increases and decreases corresponding with the species' migratory pattern [62]. Levels further showed differences among sites, which were probably driven in part by both variations in localized whale densities and differing sound propagation conditions, and varied among years as a result of fluctuations in population abundance [60,97]. In general, humpback whale singers throughout the entire survey area and independent from any seasonal effects appear to show increased activity during crepuscular periods, reflecting times when presumably the highest total numbers of singers are present. In addition, we saw varied significant diel patterns on all recorders with average fluctuations of 3 to 4 dB, reflecting a change in the ambient noise of over 25%. Increasing noise levels in the humpback whale frequency band at night off West Maui were first reported by Au *et al.* [57]. By contrast, chorusing levels increased slightly during the day at a deep recording site off Kaua‘i [63]. We were able to show that these opposing trends are correlated and that chorusing levels increased during the day at our deep offshore Maui6 site while concurrently decreasing at the four shallow, nearshore and mid-channel sites, with the mid-channel Maui7 seeing the greatest relative difference of chorusing levels between day and night, reflecting changes in the area of highest whale densities (cf. [62]). Chorusing levels at Maui6 then rapidly dropped around sunset, while levels at the shallow sites peaked around that same time before remaining fairly constant, but high, for the rest of the night. Daytime decreases in singing activity, including the number of singers [69,100–102], have been proposed to be the result of a switch in male behavioural strategy from singing to physical competition for access to females, which is assumed to rely on daytime visibility [57]. The observed increases in chorusing levels around dawn and dusk across all our recording sites seem to lend some support to this hypothesis. Competitive behaviour among males has previously been shown to peak around midday to mid-afternoon [107], which coincides with the observed decrease in chorusing close to shore. Competitive pods are often found in deeper waters than other social groups [83,87]. In fact, Ersts & Rosenbaum [87] found that in Madagascar, only competitive pods and dyads, but not calf pods and singletons (single individual whales of unknown sex or singing behaviour) moved further away from shore into deeper waters during midday hours and proposed that this might reflect a need for deeper waters that allow greater movement within the water column. We found that larger whale groups without a calf consistently occupied deeper waters further away from shore with no diurnal changes, while singletons and dyads appear to move away from shore into deeper waters by early afternoon. Nevertheless, the increase in chorusing we observed on our offshore site suggests that a nearshore daytime decrease in chorusing is not solely the result of males switching their mating strategy from singing to competing.

Following the initial decrease in chorusing in the morning to midday, levels at the nearshore shallow Olowalu and mid-channel Maui7 sites gradually started increasing in the early afternoon hours, while levels at the Pali and the deeper Olowalu sites remained low until around sunset. It is possible that these patterns may indicate a progressive increase in overall numbers of singers as some males switch back to singing from a predominantly competitive strategy and/or resting behaviour. However, we saw a similar gradual daytime increase outside the whale season at Olowalu (electronic supplementary material, figure S4), which is associated with low-frequency reef fish sounds ([59,128]; electronic supplementary material, figure S1) and wind noise arising from wind patterns off West Maui that typically strengthen through the afternoon. During times when humpback whale chorusing becomes less dominant, such as during the beginning and end of the whale season as well as intermittently during the day amidst the peak of the season, these environmental sounds become more influential of the immediate soundscape around the recorders.

### 4.2. Behaviour and occurrence of individual singers

Nearshore singers localized using DASARs increased their distance to shore during the day. Together with our findings from the visual land-based surveys that singletons and dyads, which typically contain the majority of singers among non-calf pods (e.g. [66,109]), also moved further away from shore as the day progresses, this suggests that some singers indeed relocated offshore during the day.

When singers remain stationary, they are usually found canted with their head facing downwards between −10 and −30 m from the surface [109,117,129–131], which has been proposed to promote song transmission. Possibly, singers also seek out deeper waters and associated features such as ledges in an effort to enhance the transmission efficiency of their song [83,98,132], thereby increasing the probability of reaching the intended receivers of the display. Our results showing persistent chorusing at the offshore Maui6 site further illustrate that deep waters seem to play an important role for male acoustic display. If vocalizing in deeper waters does indeed provide acoustic advantages to singers, interference from masking, such as which might occur with the biological evening chorus (see below) off Maui—and other humpback whale breeding habitats—could be a driver impacting males’ behavioural and habitat choices. Future studies using behavioural focal-follow or tagging efforts focusing on who sings in deep waters and whether there are differences in age and/or size, as well as what potential listeners, if any, are in the proximity, may help to better understand these patterns and their ultimate causes.

Travelling while singing has been reported previously [67,98,99,109,114,133–135], but was considered rare compared with stationary singers [109,117,129–131]. We found that singers travel more frequently than previously suspected and that increasing distance to shore seems to promote travelling. Singers close to shore switched frequently between stationary singing and travelling through midday. This suggests that although singers may prefer to remain immobile while singing, there appears to be pressure to move more during those hours.

### 4.3. Availability of acoustic space as a possible driver for singer distribution on a high-density breeding ground

Conflicting findings among different studies on small-scale habitat use may results from the density-dependence of behaviours, particularly of singers. Like most humpback whale populations, the Hawai‘i Distinct Population Segment has been recovering from intense twentieth-century commercial whaling, growing at an annual rate of approximately 6–7% with an estimated abundance in the mid-2000s of over 10 000 individuals [49,52,55,136]. Consequently, whale densities on the Hawaiian breeding ground are now substantially greater than during studies conducted one or two decades ago (e.g. [96,98,106]; cf. [137] for recent density estimates from line-transect vessel surveys off West Maui). Densities in Hawai‘i are also greater than on many other breeding grounds (e.g. [58,84,138–143]; also cf. [144]).

Frankel *et al.* [98] found that singers off Hawai‘i island consistently spaced themselves by approximately 5 km and suggested that such spatial separation among singers serves to reduce acoustic interference from one another. Singers in our study were spaced at a median of just under 2 km despite surveys taking place towards the end of the season when overall whale densities are lower (cf. [53,55,137,145]). This is considerably closer than previously reported off Hawai‘i island [98], Maui [105] and a Mexican breeding ground [58], and similar to distances reported for non-singing singletons [98]. Our results suggest that singing activity in Hawai‘i may at least partly be influenced by the space available for singers to spatially separate and is potentially both density-dependent and density-limited. A possible limiting effect of whale density on singing activity has also been reported by Kügler *et al.* [62] who found that singer chorusing levels are strongly correlated with local whale abundance only up to a threshold, after which levels plateaued or even decreased with further increasing whale densities. We therefore propose that throughout the day when whale densities are high, at least some singers move offshore to spread out and increase their distances to other singers. This is further supported by our finding that the opposing diel patterns in humpback whale chorusing between deep and shallow sites appear to be strongest when local whale densities are high, and more varied towards the beginning and end of the season (electronic supplementary material, figure S3).

### 4.4. Trends in environmental noises: do singers experience acoustic interference?

On all our nearshore recording sites, noise levels in the 1.56–3.12 kHz frequency band temporarily increased by 2 to 3 dB around sunset. This increase is tied to diel patterns in activity of snapping shrimps (Alpheidae), which are a ubiquitous source of ambient noise over 2 kHz on Hawai‘i's reefs as well as shallow areas [146] (figure 3), and sunset peaks are well documented (e.g. [59,110,147]). By contrast, a similar sunset peak at the offshore Maui6 site temporarily raised the noise floor almost 30-fold (by over 15 dB; figures 2, 3 and 6*b*). This non-humpback whale ‘biological evening chorus' has been observed by the authors of this study and documented by Lammers *et al.* [148] in recordings from various locations throughout the Hawaiian archipelago, and is presumed to be linked to the vertically and horizontally migrating mesopelagic boundary layer associated with island slopes [149]. Similar evening choruses with peak energy approximately 2 kHz have also been found in other regions [150–152]. Thus, this chorus represents a widespread but still poorly understood biological and acoustic phenomenon that influences the marine soundscape in Hawaiian waters and beyond.

The majority of fundamental frequencies of humpback whale song are produced below 2 kHz, but higher peak frequencies of individual units are known and harmonics can extend to at least 24 kHz [117,153]. We also saw this influence of song in frequencies above the 1.5 kHz limit selected for our analysis during times of high humpback whale chorusing intensity (figures 2 and 6). Although an audiogram has not yet been produced for humpback whales, mammals are generally considered to be able to hear sounds in the frequencies they produce [65]. There is indication that song characteristics, including peak frequency of units, may help convey fitness information of the singer to other males and/or females [68–70].

Acoustic masking, the elevation of the audibility threshold for vocalizations through the increase of the ambient noise floor and therefore the reduction of the communication space, has been receiving growing attention due to increasing anthropogenic noise in the ocean (e.g. [154–158]), but can also result from natural sound sources such as wind, precipitation or acoustic signals from other species [156,158]. Many mammals adjust their vocalization amplitude (source level) with increasing ambient noise [159]. This behavioural response, known as the ‘Lombard effect’, has been found in other cetacean species, including belugas (*Delphinapterus leucas*; [160]), orcas (*Orcinus orca*; [161,162]), North Pacific minke whales (*Balaenoptera acutorostrata*; [163]), North Atlantic right whales (*Eubalaena glacialis*; [164]), and bowhead whales (*Balaena mysticetus*; [165]). A Lombard effect has also been shown to occur for social calls from humpback whales on an Alaskan feeding ground [166] and along the East Australian migration route [167]. By contrast, male singers appear to change the structure and ultimately the duration of their song [155,168] or cease singing altogether [169,170] in response to increased noise instead of raising their source levels. Recent studies conducted off Kaua‘i, Hawai‘i [171] and along the East Australian migration route [172] found some evidence of an increase in source levels for song vocalizations with increasing ambient noise levels. However, the magnitude of the Lombard response on the Hawaiian breeding ground was markedly lower than those found off Australia, and generally in other (marine) mammals. Guazzo *et al.* [171] concluded the singers do not fully compensate for increasing ambient noise, thus leaving their communication space reduced by high noise and Girola *et al.* [172] proposed this may be because in Hawai‘i singers possibly already sing close to the maximum possible levels for the species, thus being more limited in their Lombard response. Given the combination of these findings, we hypothesize that singers off Maui move back inshore at night to avoid masking from the loud offshore non-humpback evening chorus. However, we acknowledge that other, yet to be determined, behavioural factors could also be influencing the movement of singers.

### 4.5. Small-scale habitat use in light of possible anthropogenic interference

There is presently no empirical data suggesting that vessel presence and vessel noise are a major driver for the observed diel patterns off West Maui. Nevertheless, a possible influence of vessel activity and anthropogenic noise on habitat use and behaviour choices of humpback whale singers (and non-singers) should receive future attention, both off Maui, which has seen an increase in small vessel traffic (cf. [173,174]) since earlier studies on vessel impacts were conducted [57,96,175], as well as other breeding grounds that are already subjected to higher levels of anthropogenic activity. In particular, singers might be impacted by vessel noise. While Au and Green [175] concluded that acoustic masking from whale watching vessels of Maui is probably minimal, studies in other regions have reported a negative effect on singers, such as in Japan [170]. A recent study from the Gulf of Tribugá, a Colombian breeding ground, indicated a possible reduction of communication space by over 60% in the presence of even just one whale watching vessel [176]. Most humpback whale populations continue to grow, and acoustic communication space may become a limiting factor for singers with increased densities, as we proposed for the high-density Maui breeding ground. Those whales might already experience some level of acoustic interference from anthropogenic noises or they might adopt changes in their spatio-temporal patterns to mitigate intraspecific masking that in turn could result in an increased exposure to anthropogenic or other high-intensity noises. The resulting combined effects of reduced communication space may then have fitness consequences, as song is presumed to play an important function for breeding.

## 5. Conclusion

The synthesis of our results using a combination of acoustic and visual methods suggests that the observed diel patterns in humpback whale chorusing off Maui are the result of opposing drivers. Singers move offshore beginning in the morning to increase their spacing, presumably in an attempt to reduce acoustic interference, and/or to seek out deeper waters for enhanced acoustic transmission. Around sunset, they then move back inshore, possibly to avoid the loud biological evening chorus, thereby increasing singer density and thus reducing spacing in shallower waters. This creates a dynamic of diel movements of singers that maximizes the efficiency of their acoustic display, and probably their reproductive success, that is repeated daily throughout the breeding season. These results provide new empirical evidence of the importance of song within the humpback whale mating system. We showed that singers to some degree appear to favour singing over switching to non-singing behaviours, such as physical competition or resting, when experiencing unfavourable conditions, including intraspecific and environmental acoustic interference, and responses may include temporary small-scale redistributions. Our findings warrant future studies on diel patterns and inshore versus offshore distribution of singers on other breeding grounds, including the other Hawaiian islands, some of which also experience the biological evening chorus, to understand whether the observed trends are unique to the Maui area or represent overarching responses to conditions that are shared across different areas.

## Data Availability

Data and relevant code for this research work are stored in GitHub: https://github.com/akuegler/Kuegler_etal_SingerDielTrends and have been archived within the Zenodo repository: https://doi.org/10.5281/zenodo.10408317 [177]. Additional tables and figures are provided in electronic supplementary material [178].
